# The development of genetic and molecular markers to register and commercialize *P* *enicillium rubens* (formerly *P* *enicillium oxalicum*) strain 212 as a biocontrol agent

**DOI:** 10.1111/1751-7915.12325

**Published:** 2015-10-15

**Authors:** Maria Villarino, Antonieta De Cal, Paloma Melgarejo, Inmaculada Larena, Eduardo A. Espeso

**Affiliations:** ^1^ SGIT‐INIADepartamento de Protección VegetalMadridSpain; ^2^ CIB‐CSICDepartamento de Biología Celular y MolecularMadridSpain

## Abstract

*P* *enicillium oxalicum* strain 212 (PO212) is an effective biocontrol agent (BCA) against a large number of economically important fungal plant pathogens. For successful registration as a BCA in Europe, PO212 must be accurately identified. In this report, we describe the use of classical genetic and molecular markers to characterize and identify PO212 in order to understand its ecological role in the environment or host. We successfully generated pyrimidine (*pyr*‐) auxotrophic mutants. In addition we also designed specific oligonucleotides for the *pyrF* gene at their untranslated regions for rapid and reliable identification and classification of strains of *P* *. oxalicum* and *P* *. rubens*, formerly *P* *. chrysogenum*. Using these DNA‐based technologies, we found that PO212 is a strain of *P* *. rubens*, and is not a strain of *P* *. oxalicum*. This work presents PO212 as the unique *P* *. rubens* strain to be described as a BCA and the information contained here serves for its registration and commercialization in Europe.

## Introduction

The soil‐borne fungus, *Penicillium oxalicum* Currie and Thom strain 212 (PO212), is an effective biocontrol agent (BCA) against a large number of economically important fungal plant pathogens that infect different horticultural crops in growth chamber, glasshouse and open‐field experiments (De Cal *et al*., 1995; 2009; Larena *et al*., 2003; Sabuquillo *et al*., 2005). PO212 is also effective against potato cyst nematodes under laboratory conditions (Martinez‐Beringola *et al*., 2013).

For successful registration of a potential BCA, the microorganism in the BCA must be accurately identified (Strauch *et al*., 2011). Accurate identification of the microorganism in a potential BCA is not an inconsequential process because taxonomic classifications are continuously revised and incorrect synonymizations are frequent (Visagie *et al*., 2014). DNA‐based technologies, such as DNA fingerprinting, and molecular markers, such as those that are based on the internal transcribed spacer (ITS) of ribosomal RNA genes, are now commonly used to detect and identify fungi (McCartney *et al*., 2003; Lievens and Thomma, 2005). According to Regulation (EC) 1107/2009 of the European Parliament and of the Council of 21 October 2009 (EU 2009), the ecological interactions of PO212 in the rhizosphere and in soil must be fully understood in order to register PO212 as a BCA in Europe. The ecological interactions between a BCA, the pathogen and the host involve a number of genes, compounds and molecular mechanisms. Accordingly, understanding these interactions necessitates developing means for detecting PO212 in the ecosystem, and our current knowledge of the cellular and molecular basis of these interactions for PO212 is lacking (Vinale *et al*., 2008). Hence, the main purpose of this investigation was to develop genetic and molecular markers for detecting PO212 in the ecosystem. To this end, we undertook a series of genetic and molecular studies to develop DNA‐based technologies for identifying, characterizing and monitoring PO212 in the environment when it is used as a BCA.

**Figure 1:**
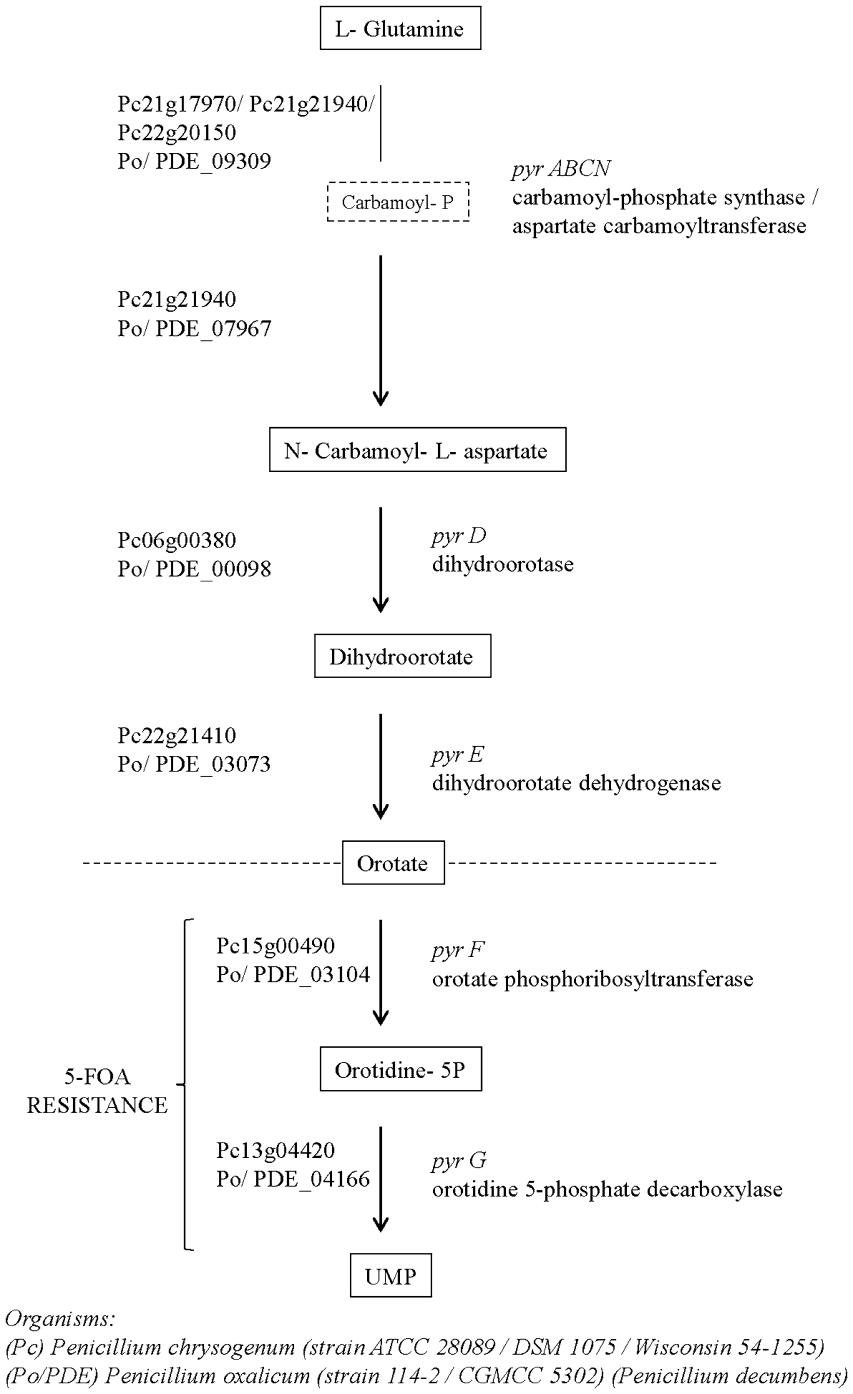
Pyrimidine biosynthetic pathway in *P* *enicillium* spp. The chart is depicting the genes and enzymes belonging to the pyrimidine biosynthetic pathway in *P* *enicillium chrysogenum* (Pc) and *P* *enicillium oxalicum* (Po/PDE). Gene designations are indicated on the left and enzyme names are on the right, and *A* *spergillus nidulans* gene designation for each step. Each of the products in this pathway is indicated in the squares. Mutations allowing 5‐FOA resistance involved the last two steps prior to UMP biosynthesis.

In transformation experiments, antibiotic and/or antifungal‐resistant genes or auxotrophic complementation are frequently used as selectable markers. Although dominant selection markers in fungi are often antifungal‐resistant genes, the permanent expression of antifungal‐resistant genes is of great concern for food‐related organisms, such as *Penicillium camemberti* (Navarrete *et al*., 2009) and for BCAs, such as PO212. Consequently, metabolic selectable markers are preferred, and the pyrimidine biosynthetic pathway is a frequently used source of such markers. In *Saccharomyces cerevisiae*, the *URA3* gene encodes for orotidine‐5′‐monophosphate decarboxylase (OMPD) (Boeke *et al*., 1987) and *URA3* gene homologues exist in filamentous fungi, such as the *pyr‐*4 gene in *Neurospora crassa* and the *pyrG* gene in *Aspergillus nidulans* and other *Penicillium* spp. (Palmer and Cove, 1975; Perkins *et al*., 1982; Díez *et al*., 1987). *ura3* or *pyrG* ^−^ mutants can be easily obtained by selecting for resistance to the toxic antimetabolite, 5‐fluoroorotic acid (5‐FOA) (Díez *et al*., 1987). The OMPD enzyme catalyses the synthesis of uridine 5′‐monophosphate (UMP) from orotidine 5′‐monophosphate (OMP) (Wittmann *et al*., 2008). The six biochemical steps of the *de novo* pathway for pyrimidine biosynthesis, which comprises the pathway to UMP, the precursor for all pyrimidine nucleotides, are conserved in all known organisms (Aleksenko *et al*., 1999; Ralli *et al*., 2007). The biosynthesis of UMP in filamentous fungi proceeds from aspartate and carbamoyl phosphate through the intermediate, orotic acid to OMP (Díez *et al*., 1987). In animals and fungi, the first two steps of the pathway are performed by a multifunctional enzyme, *pyrABCN*, which comprises the activities of carbamoyl phosphate synthetase (CPSase) and aspartate transcarbamylase (ATCase) (Aleksenko *et al*., 1999). The last steps of the pathway are performed by the following enzymes: dihydroorotase (DHOase, *pyrD*, gene designation follows the *A. nidulans* nomenclature), dihydroorotate dehydrogenase (DHOdehase, *pyrE*), orotate phosphoribosyltransferase (OPRTase, *pyrF*) and orotidine 5′‐monophosphate decarboxylase (OMPdecase, *pyrG*) (Fig. 1). Pyrimidine auxotrophic orotidine‐5′‐phosphate decarboxylase mutants of several fungi, such as *S. cerevisiae* (Boeke *et al*., 1984), *Podospora anserina* (Boeke *et al*., 1984; Razanamparany and Begueret, 1986), *Penicillium chrysogenum* (Díez *et al*., 1987), *Aspergillus niger* (Goosen *et al*., 1987) and *Trichoderma reesei* (Berges and Barreau, 1991), have been isolated by screening for resistance to 5‐FOA (Fig. 1).

Although DNA‐based technologies have been used to detect and identify *Penicillium* spp. in various commercial areas, such as the food industry (Navarrete *et al*., 2009), these technologies have not been developed for identifying PO212 when it is used as a BCA. Hence, the secondary objectives of the investigation were (i) to obtain *pyr*‐ mutants of PO212 in order to study the *in vitro* interactions between PO212, the pathogen and the host; (ii) to develop a molecular technique for accurately detecting and identifying PO212; and (iii) to identify different *Penicillium* spp. in field samples using a *pyr* gene as a molecular marker.

## Results

### Isolation of 5‐FOA‐resistant PO212 mutants

First, we analysed the sensitivity of PO212 to 5‐FOA. PO212 did not grow on potato dextrose agar (PDA) or *A. nidulans* minimal medium (MMA) supplemented with 4 mg ml^−1^ of 5‐FOA after 4 days, but the growth of PO212 was restricted when supplemented with 1.5 and 2 mg ml^−1^ of 5‐FOA. Thus, we used the last conditions to isolate *pyr*‐ mutants. Twenty putative *pyr*‐ mutants of PO212 were obtained on MMA that was supplemented with 1.5–2 mg ml^−1^ of 5‐FOA, 1.22 mg ml^−1^ of uridine and 0.56 mg ml^−1^ of uracil after 7 days of incubation at 22–25°C. Twelve of the 20 5‐FOA‐resistant PO212 colonies were found to be uridine/uracil auxotrophs.

### Sequence analysis of the CDS of the *pyr* *F* and *pyr* *G* genes

The most common mutations in the pyrimidine biosynthetic pathway that confer resistance to 5‐FOA are described in the *pyrF* and *pyrG* genes (Campuzano *et al*., 1993). Consequently, we proceeded to identify such mutations by sequence analysis of the polymerase chain reaction (PCR)‐amplified fragments that corresponded to the CDS of the *pyrF* and *pyrG* genes of PO212. For this purpose, we relied on the nucleotide sequence of the *pyrF* homologue, PDE_03104, which was recently deposited in the genome database of *P. oxalicum* strain 114‐2 (Liu *et al*., 2013), to generate specific oligonucleotides, complementary to sequences at untranslated regions (UTRs), for amplifying the complete CDS of the *pyrF* gene (Po*‐pyrF*) (Fig. 1 and Table 2). We were unable to amplify any fragment by PCR using the Po*‐pyrF* primers and genomic DNA (gDNA) from PO212. However, we were able to amplify a fragment using gDNA from strain PO6 (*P. oxalicum* strain DAOM213171; Table 1). Interestingly, the nucleotide sequence of ITS1‐rDNA5.8S‐ITS2 regions of the rDNA of PO212, which is deposited in GenBank database (entry EF103449), was similar to that of *P. chrysogenum/rubens* and other closely related species. Hence, we decided to design specific oligonucleotides from the reference genome of *P. rubens* Wisconsin strain 54–1255 (van den Berg *et al*., 2008) for amplifying a *P. chrysogenum pyrF* homologue (Pc*‐pyrF*, Pc15g00490, Table 2) by PCR. To this end, a DNA fragment (860 bp) of gDNA of PO212 was first amplified using specific oligonucleotides that were complementary to the *Pc‐pyrF* gene, and its nucleotide sequence was then compared with the genome of *P. rubens* Wisconsin strain 54–1255. We found that the sequence of this fragment was identical to that of the *Pc15g00490* gene (738 nucleotides, Fig. S1) that encodes a protein of 245 amino acids with putative OPRTase activity.

**Table 1:** Culture collection number, source, origin and GenBank accession number of the 28 *P* *enicillium* strains used in the investigation

Isolate	Culture collection	Source	Origin	GenBank accession no.
PO212	ATCC 201888	Soil	Spain	EF103449
PO1	CBS 300.97	Soil	Slovenia	EF103450
PO2	UAMH 5148	Poultry feed	Australia	EF103451
PO3	IMI 253788	Air	Spain	EF103452
PO4	DAOM 192259	Foam insulation	Canada	EF103453
PO5	DAOM 213268	Stored seeds	Canada	EF103454
PO6	DAOM 213171	Cucumber cankers	Canada	EF103455
PO7	DAOM 214729	Old cucumber roots	Canada	EF103456
PO8	ATCC 16501	Soil	Mexico	EF103457
PO9	ATCC 22095	Maize	South Africa	EF103458
PO10	IMI 112755	Rhizosphere of *Vicia faba*	Egypt	EF103459
PO11	IMI 093376	Rhizosphere of *Cicer*	India	EF103460
PO12	CBS 838.96	Purple Shiso	Netherlands	EF103461
PO13	…	Stored tobacco	Spain	EF103462
PO15	ATCC 34885	PDA contaminant	California, USA	KR233455
PO16	S53	Soil	Spain^a^	KR233456
PO17	S73	Soil	Spain^a^	KR233457
PO18	S62	Soil	Spain^a^	KR233458
PO19	S27	Soil	Spain^b^	KR233459
PO20	S56	Soil	Spain^a^	KR233460
PO21	S17	Soil	Spain^a^	KR233461
PO22	S49	Soil	Spain^b^	KR233462
PO23	S59	Soil	Spain^a^	KR233463
PO24	S60	Soil	Spain^a^	KR233464
PO25	S71	Soil	Spain^a^	KR233465
PO26	S63	Soil	Spain^a^	KR233466
PO27	573	Soil	Spain	KR233467
PO28	A1	Soil	Spain^c^	KR233468

a Location: Navalmanzano, Segovia, Spain.

b Location: Vinaderos, Avila, Spain.

c Location: Aranjuez, Madrid, Spain.

**Table 2:** List and nucleotide sequence of the primers used in this work

Primer code	Sequence (5′‐3′)
Pc pyrD 1	GCAAAAAGTGAAGATCGAC
Pc pyrD 2	GTAAGAGGATGTGCATGTG
Pc pyrE 1	TAATCGCCGTATAGGTTCG
Pc pyrE 2	CAGGATCTATCAAAGACCG
Pc pyrF 1	GACTCTTTGACTCTTTGAC
Pc pyrF 2	TCCATCCTGTTGTCTTTGC
Po114‐2 PyrF 1	ACCTCCGACTTCGTTGTCGC
Po114‐2 PyrF 2	AGTCGAGTCCTTGTTCCCTCG
Pc pyrG 1	GCCATGTCCTCCAAGTCGC
Pc pyrG 2	CTCCTATTGCGCACCCACGC
ITS5	GGAAGTAAAAGTCGTAACAAGG
ITS4	TCCTCCGCTTATTGATATGC
ITS1	TCCGTAGGTGAACTTGCGG
ITS2	GCTGCGTTCTTCATCGATGC
ITS3	GCATCGATGAAGAACGCAGC
BOX‐A1R	CTACGGCAAGGCGACGCTGACG
REP‐1R	IIIICGICGICATCIGGC
REP‐2R	ICGICTTATCIGGCCTAC

I, inosine.

The nucleotide sequence of the CDS of the *pyrG* gene from *P. rubens* Wisconsin strain 54–1255 is 831 bp long (GenBank accession number 211583497), and encodes OMPdecase (276 amino acids). Therefore, we amplified the nucleotide sequence of the CDS of the *pyrG* gene using gDNA of PO212 and specific oligonucleotides (Table 2). The nucleotide sequence of the CDS of the *pyrG* gene of PO212 was identical to that of the *pyrG* gene of *P. rubens* Wisconsin 54–1255 (Pc13g04420, Fig. S2). We used the same strategy to amplify, identify and characterize the nucleotide sequence of the CDS of the *pyrD* and *pyrE* genes of PO212. We then compared the nucleotide sequences of the CDS of these PO212 genes and the orthologues of *P. rubens* Wisconsin 54–1255 (Pc06g00380 and Pc22g21410, Fig. S3 and S4), and the results of this sequencing analysis revealed that PO212 is very closely related to *P. rubens*.

**Figure 2:**
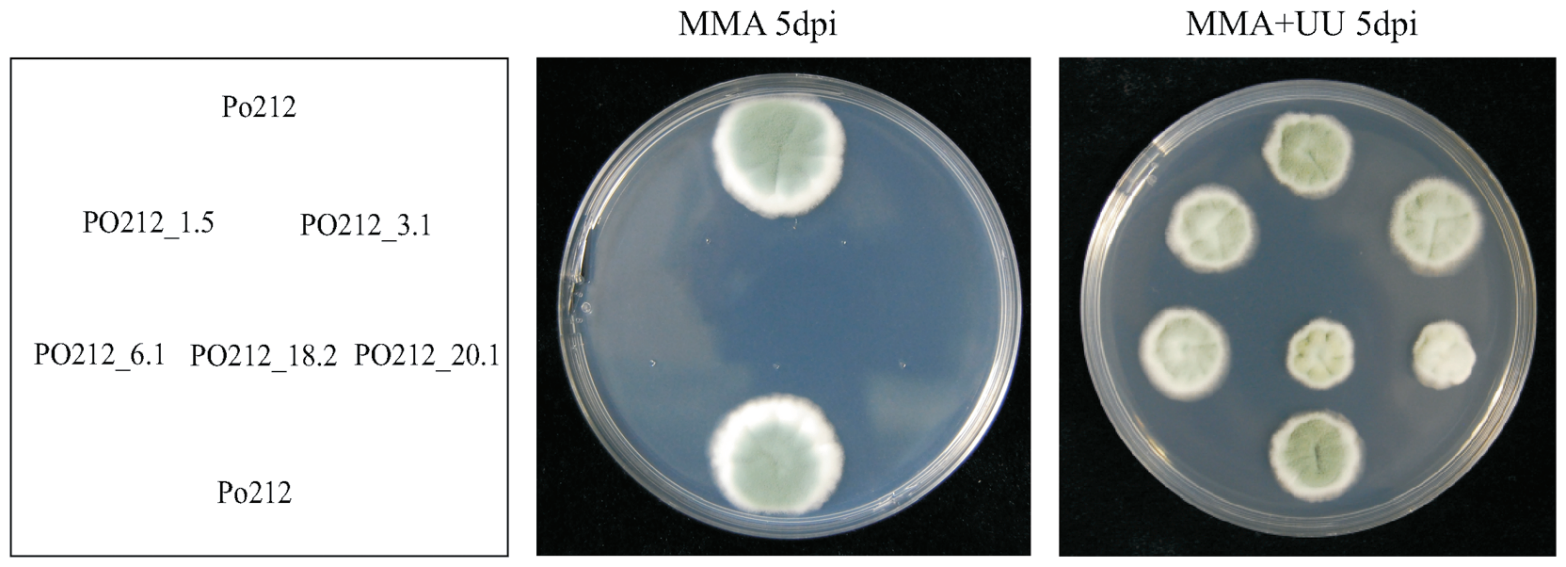
The growth of *P* *enicillium* strain PO212 and *pyr*‐ mutants of PO212 on MMA with (+ UU) or without the addition of 0.56 mg ml^−1^ of uracil and 1.22 mg ml^−1^ of uridine at 20–25°C for 5 days. dpi, days post inoculation.

Next we sequenced the CDS of the *pyrG* gene of each of the five pyrimidine auxotrophic PO212 mutants. We detected changes in the nucleotide sequence of the CDS of the *pyrG* gene of three *pyr*‐ mutants. In the two remaining *pyr*‐ mutants in which *pyrG* sequence was unaltered, we then amplified and sequenced the CDS of their respective *pyrF* gene. Nucleotide changes were then found in the CDS of the *pyrF* gene in those two *pyr*‐ mutants. Mutations found and the derived amino acid substitutions of *pyr*‐ mutants of PO212 are shown in Table 3. Except for the PO212_18.2 mutant that carries a mutation in the *pyrG* gene that causes a truncation of PyrG protein at amino acid 104, the rest were missense mutations (Table 3). For mutant PO212_3.1, the mutation changes the initiation methionine codon of PyrF to a codon for isoleucine (Table 3), and this mutation probably renders a null allele because translation of *pyrF* mRNA must be affected. Because all mutants are indistinguishable from each other with respect to *pyr* auxotrophy and they strictly require supplementation with pyrimidines for growth (Fig. 2), we concluded that these *pyr‐* mutants of PO212 carry complete loss of function.

**Table 3:** List of 5‐fluoroorotic acid‐resistant mutants of *P* *enicillium* strain PO212 isolated in this work

Mutant strain	Gene	DNA mutation	Change in protein
PO212_1.5	*pyrF*	G377A	R126H
PO212_3.1	*pyrF*	G3A	M1I
PO212_6.1	*pyrG*	T851G	W266G
PO212_18.2	*pyrG*	C365T	Q104stop
PO212_20.1	*pyrG*	T513G	L153R

### Sequence analysis of the ITS1‐5.8S‐ITS2 regions

**Figure 3:**
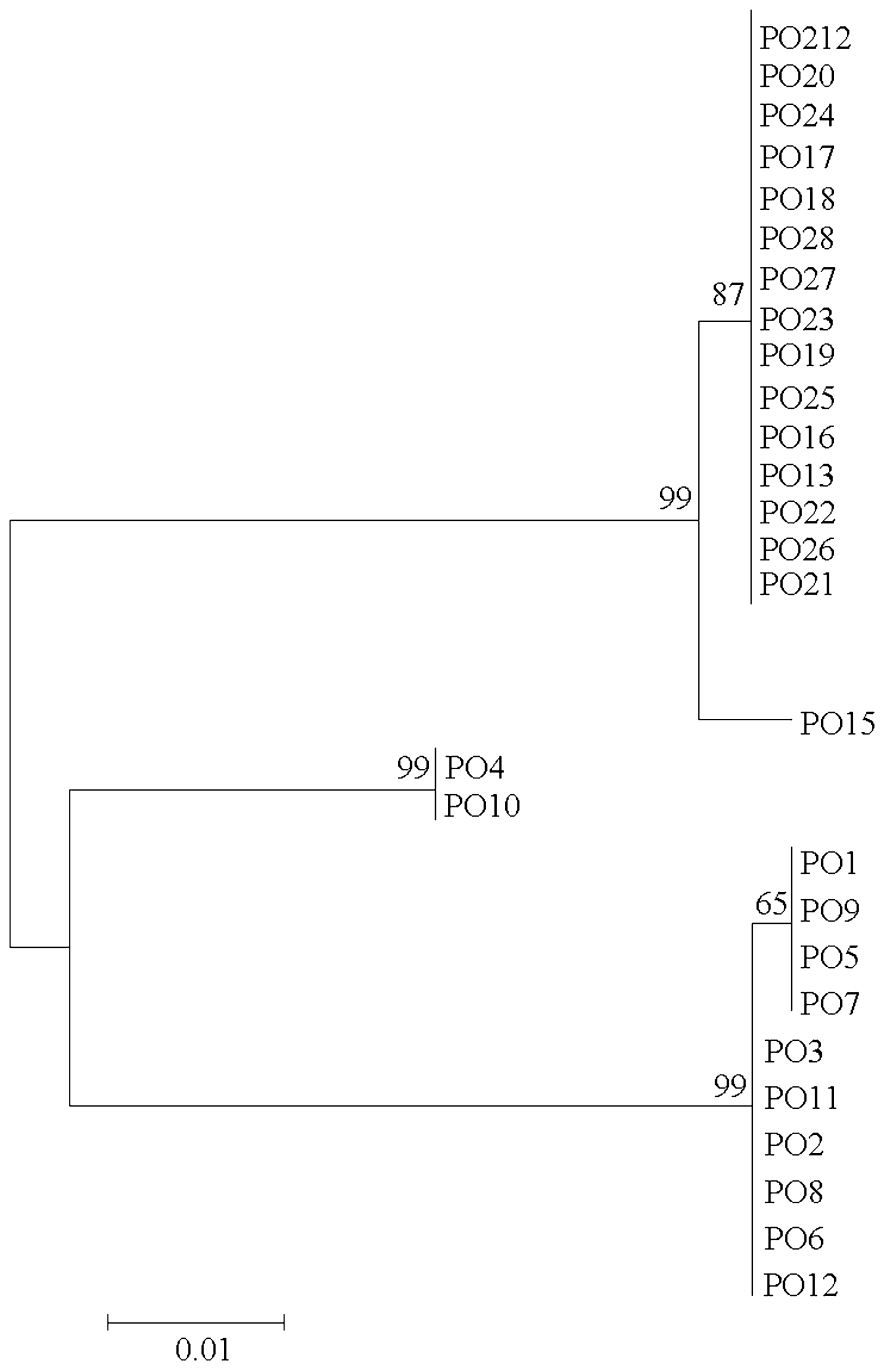
Dendrogram of the phylogenetic relationship between *Penicillium* strain 212 (PO212) and the 27 *P* *enicillium* isolates. The dendrogram was based on the nucleotide sequences of the ITS1‐5.8S‐ITS2 regions of the ribosomal RNA genes, and constructed by MEGA (version 5.2; http://www.megasoftware.net/) using the neighbour‐joining method and the Jukes–Cantor model. Numbers at each node indicate the percentage of bootstrap samples of 1000 replicates that are supported in the cluster on the right (only values > 70% are shown for clarity).

Because the nucleotide sequences of the *pyrF* and *pyrG* genes of PO212 were identical to those of the *P. rubens* homologues, we questioned whether the previous morphological classifications of the *P. oxalicum* isolates were accurate. Consequently, we amplified and then sequenced the ITS1‐5.8S‐ITS2 regions of the rDNA gene from the 28 *Penicillium* strains (Table 2). The nucleotide sequences of the ITS1, 5.8S and ITS2 regions were then compared with that of PO212, and a dendrogram that was based on the pairwise comparison of the nucleotide sequences of the ITS1‐5.8S‐ITS2 regions of PO212 and the 27 *Penicillium* strains was constructed. Two distinct groups were identified with a mean level of similarity of 99% (Fig. 3). Most of the Spanish *Penicillium* isolates clustered in one clade with PO15 isolate from the United States. The other clade comprised the non‐Spanish *Penicillium* isolates except PO3, with PO4 and PO10 being the most divergent isolates (Fig. 3).

The nucleotide sequences of the ITS1‐5.8S‐ITS2 regions of the rDNA gene of PO212 and the 27 *Penicillium* strains were also compared using the Lalign local alignment program (http://www.ch.embnet.org/software/LALIGN_form.html). Conservation between the nucleotide sequence of the ITS1‐5.8S‐ITS2 regions of PO212 and that of the Spanish *Penicillium* strains is 100%. This conservation is reduced to 93–94% when the nucleotide sequence of the ITS1‐5.8S‐ITS2 regions of PO212 is compared with that of the non‐Spanish *Penicillium* strains.

### Analysis of the BOX and repetitive extragenic palindromic (REP) DNA fingerprints

**Figure 4:**
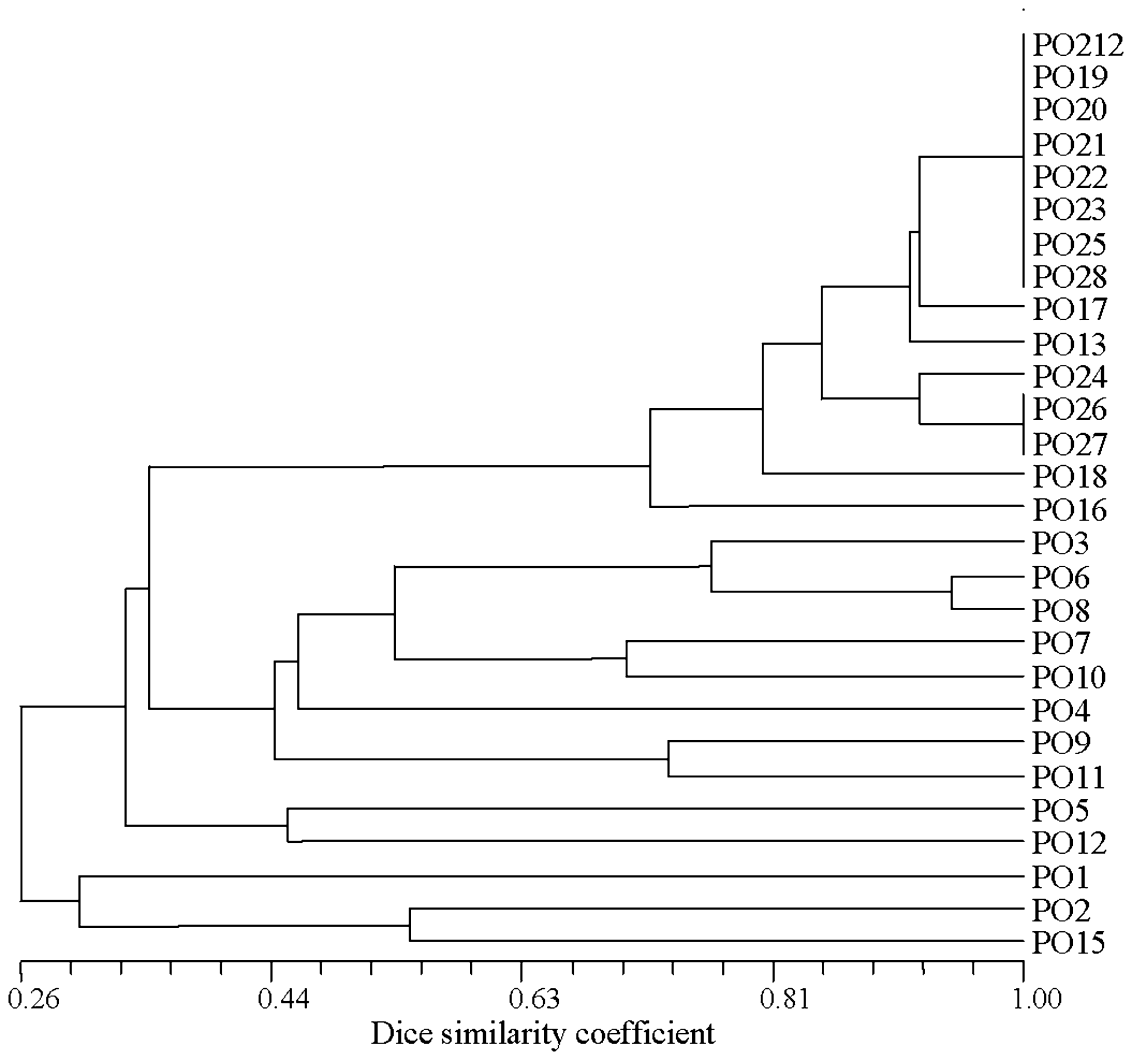
Dendrogram of the phylogenetic relationship between *Penicillium* strain PO212 and the 27 *P* *enicillium* isolates. The dendrogram was based on BOX DNA fingerprints, and constructed by first calculating Dice coefficients of similarity for pairs of isolates using the NTSYS‐pc version 2.10b software package (Exeter Software, Setauket, NY, USA) and then converting the similarity matrices into a dendrogram using the unweighted pair group method with arithmetic average method and the sequential, agglomerative, hierarchical and nested clustering program of NTSYS‐pc version 2.10b software package.

**Figure 5:**
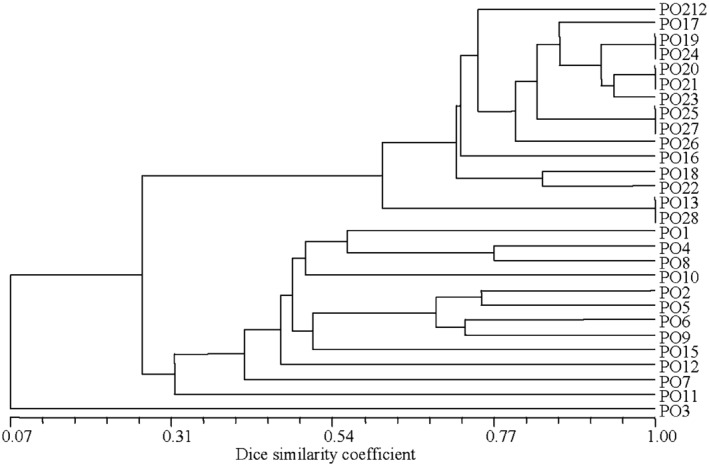
Dendrogram of the phylogenetic relationship between *P* *enicillium* strain PO212 and the 27 *P* *enicillium* isolates. The dendrogram was based on REP DNA fingerprints, and constructed by first calculating Dice coefficients of similarity for pairs of isolates using the NTSYS‐pc version 2.10b software package (Exeter Software, Setauket, NY, USA), and then converting the similarity matrices into a dendrogram using the unweighted pair group method with arithmetic average method and the sequential, agglomerative, hierarchical and nested clustering program of NTSYS‐pc version 2.10b software package.

The DNA fingerprints of PO212 and the 27 *Penicillium* strains were obtained by amplifying the BOX elements and the REP sequences in their gDNA. The sizes of all DNA fingerprints were between 100 and 1200 bp. Dendrograms of the phylogenetic relationship between PO212 and the 27 *Penicillium* strains that were based on the BOX and REP DNA fingerprints were similar to those that were based on the ITS markers: the Spanish *Penicillium* strains were distinctly different from the non‐Spanish *Penicillium* strains (Figs 4 and 5). The dendrograms of the phylogenetic relationship that were based on the BOX DNA fingerprints revealed that the PO3 strain clustered in the clade that comprised the non‐Spanish *Penicillium* strains. Interestingly, examination of the dendrograms of the phylogenetic relationship that were based on the REP DNA fingerprints revealed that the PO3 strain did not cluster in the clade of either the Spanish or non‐Spanish *Penicillium* strains.

### Using the *pyr* *F* gene to classify *P* *enicillium* strains

Because we could use the specific primers that we generated for amplifying the *pyrF* genes from either *P. rubens* (Pc‐*pyrF*) or *P. oxalicum* (Po‐*pyrF*), we reasoned that these primers could also be used to accurately classify all Spanish *Penicillium* strains and selected non‐Spanish *Penicillium* strains (PO1 from Slovenia, PO6 and PO7 from Canada and PO12 from the Netherlands).

PCR products were obtained from gDNA of the Spanish *Penicillium* isolates when using the Pc*‐pyrF* oligonucleotides. We also obtained PCR products when we amplified the *pyrF* gene of PO26 using the primer pairs, Pc‐*pyrF* or Po‐*pyrF*. In contrast, PCR products were not obtained when using gDNA from PO3 and the primer pairs, Pc‐*pyrF* or Po‐*pyrF*. We attributed this failure to amplify the gDNA of PO3 to the low similarity of this Spanish *Penicillium* isolate with either the Spanish or non‐Spanish *Penicillium* isolates. Furthermore, this finding of a low similarity is consistent with our previous finding that PO3 is closely related to *Penicillium corylophilum* (entry EF103452) in dendrograms that are based on the nucleotide sequences of the ITS1‐5.8S‐ITS2 regions.

The American and European *Penicillium* isolates, PO1, PO6, PO7 and PO12, were originally classified as *P. oxalicum* species according to the nucleotide sequence of their ITS1‐5.8S‐ITS2 regions. We were also able to obtain PCR products when we amplified the *pyrF* gene of PO1, PO6, PO7 and PO12 using specific Po*‐pyrF* oligonucleotides. These results verify the use of Pc‐*pyrF* and Po‐*pyrF* as primers for amplifying the *pyrF* gene of *P. rubens* and *P. oxalicum* in order to reclassify *Penicillium* strains in laboratory collections.

Multiple sequence alignment of the CDS of the *pyrF* gene of PO212 and the 27 *Penicillium* strains revealed a high level of conservation of the nucleotide sequence of the CDS of the *pyrF* gene among the *pyrF* homologues (Fig. S1). Examination of the dendrogram that is based on the nucleotide sequence of the CDS region of the *pyrF* gene also revealed that PO212 is a strain of *P. rubens*. Examination of this dendrogram also revealed strong divergence between PO212 and *P. digitatum* and *P. glabrum*. In fact, the nucleotide sequence of the *pyrF* gene of *Aspergillus clavatus*, which is used to root the phylogenetic tree, revealed that *A. clavatus* is more closely related to the other *Penicillium* spp. than to *P. oxalicum* (Fig. S1). This strong conservation in the nucleotide sequences of the CDS of the *pyrF* gene, together with the presence of the macro‐ and micromorphological characteristic of the *Penicillium* strains, strongly suggests that the nucleotide sequences of the CDS of the *pyrF* gene can be used to detect and accurately identify *Penicillium* isolates.

## Discussion

The evaluation and authorization of plant protection products that contain microorganisms are regulated in the European Union (EU) by Regulation 546/2011 (EU 2011). According to these regulations, the identity of a microorganism should be clearly stated for placing of plant protection products on the market. Previous taxonomic classifications of the BCA PO212 and the soil‐borne Spanish isolates identified these isolates as strains of *P. oxalicum*, and this identity was based on colour, size and shape of the colony and the various characteristics, such as conidial size and conidiophore morphology (Ramirez, 1982). Using these morphological and phenotypic characteristics to identify *P. oxalicum* strains can be challenging, and contradictory findings and grouping of more than one species often occur (Taylor *et al*., 2000). Species in the *Penicillium* genus exhibit only few distinguishing features, which additionally may vary depending on the growth conditions and the culture medium (Henk *et al*., 2011). Taxonomic classification in the genus *Penicillium* is challenging because it is being continually reviewed. This ongoing review stems from the genetic diversity of this genus that has been unmasked by random amplified polymorphic DNA markers and nucleotide sequencing and restriction fragment length polymorphism markers of the ITS1‐5.8S‐ITS2 regions of their rDNA (Dupont *et al*., 1999; Tiwari *et al*., 2011). For example, the reference fungus of the genus *Penicillium*, *P. chrysogenum*, has recently been reclassified (Houbraken *et al*., 2011) following a phylogenetic analysis of numerous *P. chrysogenum* isolates. The results of this analysis revealed the presence of two highly supported clades that represent two species: *P. chrysogenum* and *P. rubens*. Even Fleming's original penicillin‐producing strain and the full genome‐sequenced strain of the filamentous fungus, *P. chrysogenum* Wisconsin 54–1255 (NRRL 1951) (van den Berg *et al*., 2008), have been re‐identified as *P. rubens*. Such reclassifications show that no strain of *P. chrysogenum sensu stricto* has had its entire genome sequenced (Houbraken *et al*., 2011). In this study, we found that well‐defined clusters in the dendrograms were based on the ITS1‐5.8S‐ITS2 regions and the BOX and REP DNA fingerprints of PO212 and the 27 *Penicillium* isolates. These phylogenetic analyses also revealed that all the Spanish *Penicillium* isolates are grouped together, suggesting that they belong to the same *Penicillium* species. Thus, the identification of a strain needs to be based on the newest available methodologies and knowledge about the species and its genus. Progress and advances in molecular and *in silico* genetics have resulted in the invention of novel genetic and molecular tools for rapid identification and accurate taxonomic classification of fungal species (Dupont *et al*., 1999).

Here we show that distinguishing between strains of *P. rubens* and *P. oxalicum* can be achieved by the use of the *pyrF* gene. Given that the pyrimidine biosynthetic pathway is a highly conserved pathway in the fungal kingdom (see below), sequence analysis of those genes that encode the enzymes in this pathway can be used to accurately classify fungal isolates. Because the size of the CDS of the *pyrF* gene of PO212 is only 738 nucleotides, it is adequate for a standardized process of PCR amplification and sequencing. Conservation of the nucleotide sequence of the CDS of the *pyrF* gene homologues is high, even at the third codon position, and this high level of conservation is most likely a consequence of the reduced size of the *pyrF* gene homologues (Fig. S1). Thus, when changes in nucleotide sequence are found, as in the case of PO212‐*pyrF* and *P. oxalicum* 114‐2 *pyrF* genes, these can be taken as evidence of variability among *Penicillium* species. Additionally, despite a high level of conservation of the nucleotide sequence of the CDS of the *pyrF* gene homologues, the level of conservation of the nucleotide sequence of the flanking UTRs is low. We found that this feature was very useful for designing the specific primers that we used for amplifying the CDS of the *pyrF* gene by PCR from either *P. rubens* or *P. oxalicum*. We consider that these *pyrF*‐specific primers would serve, as those previously designed for other phylogenetic markers (e.g. β‐tubulin, calmodulin, RNA polymerase), to achieve with accuracy the identification of these two fungal species at any global location.

Furthermore, identification of *pyrG* and *pyrF* gene homologues of PO212 prompted us to revise the taxonomic classification of PO212 and these other strains in our laboratory collection. As a result of this examination, we concluded that many of naturally occurring isolates from diverse Spanish locations, which were initially classified as *P. oxalicum* using standard taxonomic clues of the genus *Penicillium*, were actually classified as *P. rubens.* Interestingly, the Center for Agricultural Bioscience International has also classified PO212 as a strain of *P. rubens/chrysogenum* using classical phylogenetic markers. This new finding that PO212 is a strain of *P. rubens/chrysogenum* is intriguing because a biocontrol activity has been shown for strains of *P. rubens*. Curiously, it has only been reported to date that dry mycelium preparations of *P. chrysogenum* (*P. rubens*), which is a waste product of the pharmaceutical industry and sometimes used as an organic fertilizer in commercial agriculture, does protect plants against fungal pathogens, such as *Fusarium oxysporum* f. sp. *vasinfectum* and *Verticillium dahliae* Kleb (Chen *et al*., 2006), and infestations of the nematode, *Meloidogyne javanica* (Gotlieb *et al*., 2003).

Finally, our work demonstrates that traditional genetic techniques, focused on the isolation of auxotrophic mutants, can be used with a field sample. We successfully isolated uridine‐ and uracil‐requiring mutants of PO212 by selecting 5‐FOA‐resistant mutants. Loss of function mutations in *pyr* genes have been extensively used in combination with molecular tools to understand numerous biological and biochemical processes in fungi (Ballance and Turner, 1985; Woloshuk *et al*., 1989). The introduction of such DNA‐based technologies will improve our current understanding of the cellular and molecular mechanisms of the interactions between PO212, phytopathogenic fungi and plants. Similar strategies to study plant–fungal interactions have been already used in other fungi (Lagopodi *et al*., 2002; Olivain *et al*., 2006). In *Penicillium* (Díez *et al*., 1987) and other fungi, mutations resulting in both pyrimidine auxotrophy and resistance to 5‐FOA locate in two independent loci (Boeke *et al*., 1984; Razanamparany and Begueret, 1986; Akileswaran *et al*., 1993; Takeno *et al*., 2004). Mutations that modify the activity of OMP‐pyrophosphorylase, encoded by the *pyrF* gene, and the activity of OMP decarboxylase, encoded by the *pyrG* gene, can be isolated and the resultant mutants exhibit an identical and non‐additive pyrimidine‐requiring phenotype. Because we were able to characterize these mutations in PO212, we were also able to demonstrate universality of this selection procedure that can be applied to other *Penicillium*.

In conclusion, we have generated auxotrophic mutants of PO212 in order to improve our current understanding of PO212 as a BCA. We have designed specific oligonucleotides of the *pyrF* gene to be used in a standard PCR that can be used to rapidly and reliably identify two species of the genus *Penicillium*, *P. rubens* and *P. oxalicum*, in field samples. Furthermore, nucleotide sequencing of the CDS of the *pyrG* and *pyrF* genes revealed that PO212 is a strain of *P. rubens*, and is not a strain of *P. oxalicum*. The availability of phylogenetic markers and nucleotide sequencing of the genome of fungi has created exciting opportunities for comparative genomics between fungal species. Additionally, their availability and genome sequencing will enable reliable and definitive classification of fungal strains at the species level within a genus. Furthermore, these technologies can be used to characterize the mode of action, the ecology and the fitness of PO212, which is also a required information for their registration and commercialization as a BCA in Europe. In the future, we plan to fully sequence the genome of PO212 and then compare its genome to full genome‐sequenced strains of *Penicillium* species. Results of all these studies will improve the efficacy and practical application of PO212 when it is used as a BCA for plant disease control.

## Experimental procedures

### *P* *enicillium* strains, growth media and culture conditions

The investigation comprised 28 different strains of *Penicillium* that were obtained from different geographical regions, habitats and culture collections (Table 1). The Spanish strains were mainly soil‐borne fungi and the non‐Spanish strains came from various hosts in other global regions. The identity of each *Penicillium* strain was confirmed using the macro‐ and micromorphological characteristics of Ramirez (Ramirez, 1982).

The 28 *Penicillium* strains were stored at −80°C in 20% glycerol (long‐term storage) and at 4°C on PDA (Difco, Detroit, MI, USA) slants in the dark (short‐term storage). The 28 *Penicillium* strains were propagated at 22–25°C on PDA, *A. nidulans* complete medium or MMA, which contained 5 mM of ammonium tartrate as a nitrogen source and D‐glucose 1% (p/v) as a carbon source (Cove, 1966). Uridine (1.22 mg ml^−1^) or uracil (0.56 mg ml^−1^) was added to the growth medium when required.

### Isolation of nucleic acids

PO212 (ATCC number 201888) was used as the source of gDNA for genome sequencing and for obtaining *pyr‐* mutants in mutagenesis experiments. Conidia from wild‐type (wt) PO212, the 27 *Penicillium* strains and the pyrimidine auxotrophic mutants of PO212 were used as the starting material for obtaining mycelia, which were then used for extracting gDNA. These conidial cultures were grown on MMA or PDA at 25°C, and the mycelia were collected by filtration. gDNA was extracted from the mycelia using our previously published protocols (Etxebeste *et al*., 2009; Larena and Melgarejo, 2009), and the gDNA samples were stored at −20°C until required.

### Strategies for obtaining 5‐FOA‐resistant mutants of PO212

The isolation of 5‐FOA‐resistant mutants of PO212 was carried out on PDA or MMA plates that were supplemented with 1–4 mg ml^−1^ of 5‐FOA (Apollo Scientific, Stockport, UK), 1.22 mg ml^−1^ of uridine and 0.56 mg ml^−1^ of uracil, and incubated at 20–25°C for 5 days. Clones that were resistant to 5‐FOA would lack either OPRTase, which is encoded by the *pyrF* gene, or OMPdecase, which is encoded by the *pyrG* gene.

The 5‐FOA‐resistant mutants were isolated, purified through two selective passes on MMA plates that were supplemented with 1–4 mg ml^−1^ of 5‐FOA and then tested for uridine auxotrophy on PDA plates with and without uridine and uracil. The *pyr*‐ mutants do not grow on MMA without uridine and uracil (Fig. 2).

### Amplification and sequence analysis of the coding region of the *pyr* genes

For amplification of the *pyr* genes, specific primers (Table 2) were designed using the genomic information of *P. oxalicum* strain 114‐2 (formerly classified as *P. decumbens*; Liu *et al*., 2013) and *P. rubens* (formerly *P. chrysogenum*) (strain ATCC 28089/DSM 1075/Wisconsin 54–1255; van den Berg *et al*., 2008) in the National Center for Biotechnology Information's database (http://www.ncbi.nlm.nih.gov/blast). The oligonucleotide pair, Pc‐*pyrF* and Po‐*pyrF*, was routinely used in the taxonomical analyses of all Spanish isolates and a selected number of non‐Spanish strains (PO1 from Slovenia, PO6 and PO7 from Canada and PO12 from Netherlands).

The PCRs were performed in a 50 μl reaction mixture that contained standard Taq‐polymerase under the following conditions: an initial denaturation step of 2 min at 94°C, followed by 25 cycles of 30 s at 94°C, 30 s at 56°C, and 1 min at 72°C, and a final extension of 3 min at 72°C. The PCR products were purified using the QIAquick PCR purification kit (Qiagen GmbH, Hilden, Germany) according to the manufacturer's instructions, and then sequenced in an automated DNA sequencer (version 3.1; BigDye® Terminator) at the sequencing services of Secugen (http://www.secugen.es; Madrid, Spain). Multiple alignments of the orthologous *pyrF* genes were carried out using ClustalW2 online facility at EBI (http://www.ebi.ac.uk/Tools/msa/clustalw2/) (Fig. S1).

### Amplification and sequence analysis of the ITS1‐5.8S‐ITS2 regions

The ITS1‐5.8S‐ITS2 regions of the rDNA of each isolate were amplified by PCR with the universal primers, ITS4 and ITS5 (White *et al*., 1990), whose sequences are shown in Table 2. The PCR conditions for these amplifications were identical to those that are described in Larena and Melgarejo (2009). The PCR products were purified using the Wizard® SV Gel and PCR Clean‐up System (Promega, Madison, WI, USA), and the purified products were then sequenced using the universal primers, ITS1, ITS2, ITS3 and ITS4 (White *et al*., 1990) (Table 2), in an automated DNA sequencer (ABI 3730, Applied Biosystems, Foster City, CA, USA) at the sequencing services of CISA‐INIA, Madrid, Spain, and the DNA Sequencing Services of Stab Vida España, Madrid, Spain. The nucleotide sequences of ITS1‐5.8S‐ITS2 regions of the rDNA of each isolate were aligned and compared using BioEdit Sequence Alignment Editor 5.0.6 (Hall, 1999) and ClustalW Multiple Sequence Alignment program (version 1.82; http://www.clustal.org/) (Thompson *et al*., 1994). The GenBank accession numbers of the nucleotide sequences from each isolate are presented in Table 2.

The nucleotide sequences of ITS1‐5.8S‐ITS2 regions of the rDNA of the 28 *Penicillium* strains were assigned to species using the basic local alignment search tool (BLAST; http://www.ncbi.nlm.nih.gov/blast). A dendrogram that was based on the nucleotide sequences of the ITS1‐5.8S‐ITS2 regions (Fig. 3) was constructed by MEGA (version 5.2; http://www.megasoftware.net/) (Kumar *et al*., 2004) using the neighbour‐joining method (Saitou and Nei, 1987) and the Jukes–Cantor model (Jukes and Cantor, 1969). The reliability of the clusters was assessed by bootstrap analysis with 1000 replicates (Felsenstein, 1985).

### Amplification of the BOX element and the REP sequence

BOX‐PCRs using the BOX‐A1R primer and REP‐PCRs using the REP‐1R and REP‐2R primers (Table 2) (Louws *et al*., 1999) were used to generate DNA fingerprints of PO212 and the 27 *Penicillium* strains. The PCR conditions were identical to those that are described in Redondo *et al*. (2009). The DNA fingerprints were then visualized in 2% agarose gels under ultraviolet light after their staining with ethidium bromide. Each PCR was carried out at least twice to verify the reproducibility of the bands and reliability of the reaction.

### Determination of the phylogenetic relationships between PO212 and other *P* *enicillium* strains

In order to determine the phylogenetic relationship between PO212 and the 27 *Penicillium* strains, their DNA fingerprints were binarized (0 = absent, 1 = present), and Dice coefficients of similarity (Sneath and Sokal, 1973) for pairs of isolates were calculated using the NTSYS‐pc software package (version 2.10b; Exeter Software, Setauket, NY, USA) (Rohlf, 2002). The resultant similarity matrices were converted into dendrograms using the unweighted pair group method with arithmetic average method and the sequential, agglomerative, hierarchical and nested clustering program of NTSYS‐pc software package (Figs 4 and 5).

## Supporting information


**Fig. S1.** Multiple alignment of nucleotide sequences of the coding region of the *pyrF* genes of *Penicillium* strain PO212, *Penicillium chrysogenum* Wisconsin 54–1255 (Pc_w, XM_002560230.1), *P. chrysogenum v.1.0* (Pc, fgenesh1_pm.11_#_35, JGI code), *P. digitatum* (Pd, PDIG_06100m.01, JGI code), *P. glabrum* (Pg, CE13497_6539, JGI code), *Aspergillus clavatus* (Ac, XM_001275418.1) and *P. oxalicum* (Po, EPS28158).

**Fig. S2.** Multiple alignment of nucleotide sequences of the coding region (genomic version) of the *pyrG* genes of *Penicillium chrysogenum (rubens)* Wisconsin 54–1255 (Pc_w, Pc13g04420), *Penicillium* strain PO212 (PO212) and *P. oxalicum* (Po, PDE_04166).

**Fig. S3.** Multiple alignment of nucleotide sequences of the coding region (genomic version) of the *pyrD* genes of *Penicillium chrysogenum (rubens)* Wisconsin 54–1255 (Pc_w, Pc06g00380), *Penicillium* strain PO212 (PO212) and *P. oxalicum* (Po, PDE_00098).

**Fig. S4.** Multiple alignment of nucleotide sequences of the coding region (genomic version) of the *pyrE* genes of *Penicillium chrysogenum (rubens)* Wisconsin 54–1255 (Pc_w, Pc22g21410), *Penicillium* strain PO212 (PO212) and *P. oxalicum* (Po, PDE_03073).
