# Supplementary figures and images for: The development of genetic and molecular markers to register and commercialize P enicillium rubens (formerly P enicillium oxalicum) strain 212 as a biocontrol agent
Source: Microb Biotechnol. 2015 Oct 15;9(1):89–99. doi: 10.1111/1751-7915.12325 (PMC4720407; doi:10.1111/1751-7915.12325)

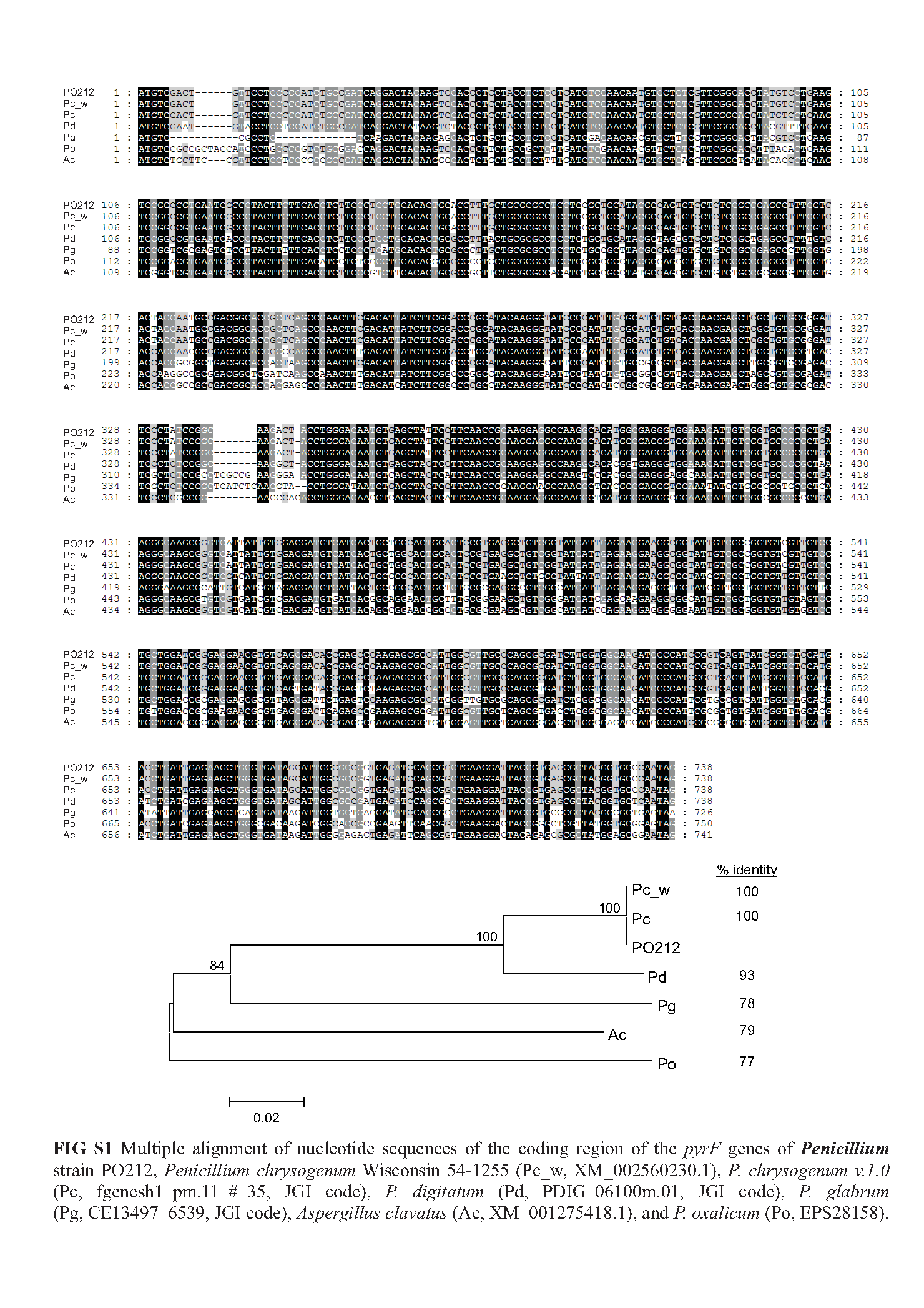

Supplement: Supplementary file 1 — Fig. S1. Multiple alignment of nucleotide sequences of the coding region of the pyrF genes of Penicillium strain PO212, Penicillium chrysogenum Wisconsin 54–1255 (Pc_w, XM_002560230.1), P. chrysogenum v.1.0 (Pc, fgenesh1_pm.11_#_35, JGI code), P. digitatum (Pd, PDIG_06100m.01, JGI code), P. glabrum (Pg, CE13497_6539, JGI code), Aspergillus clavatus (Ac, XM_001275418.1) and P. oxalicum (Po, EPS28158). [file MBT2-9-089-s001.tif]

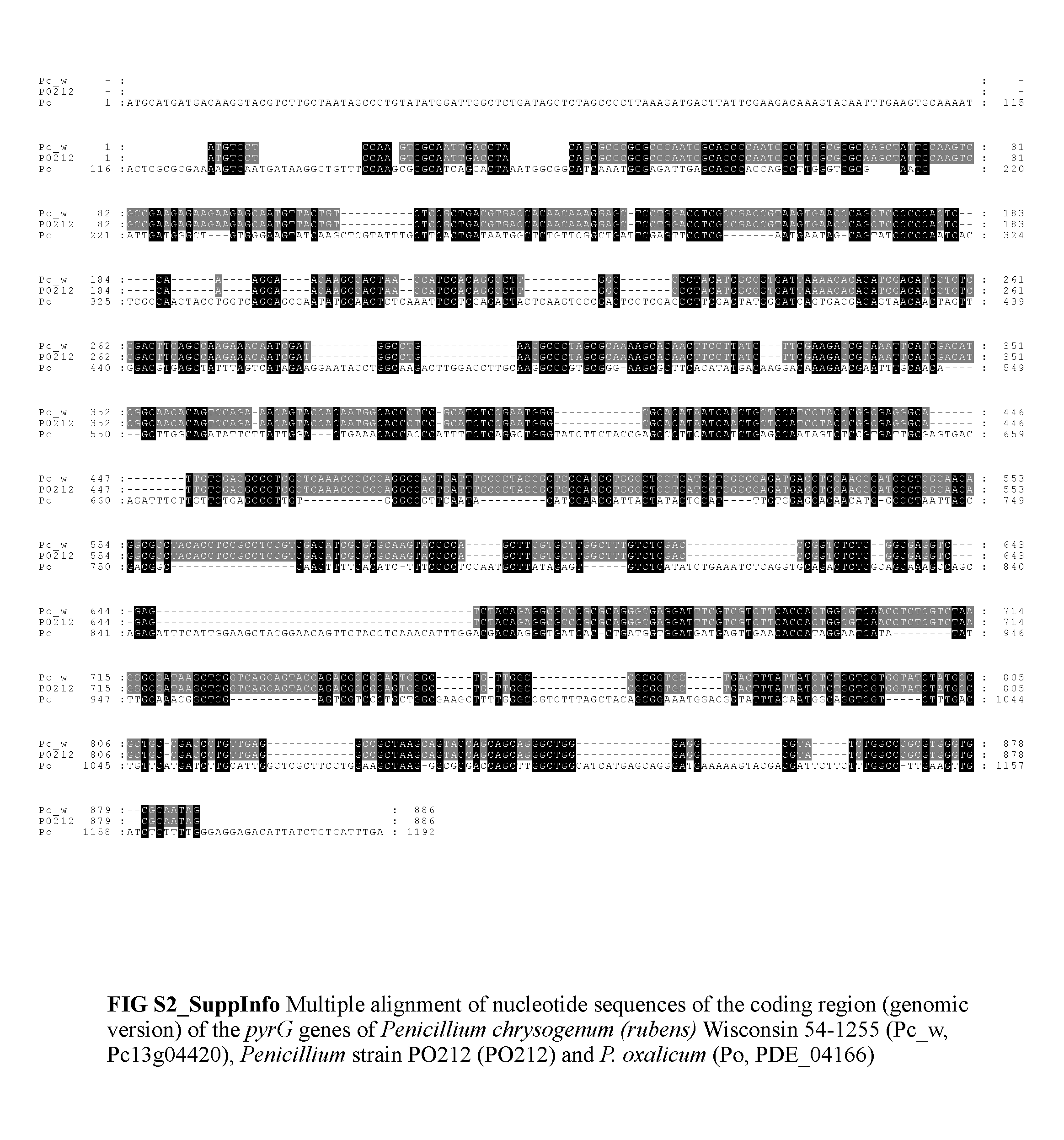

Supplement: Supplementary file 2 — Fig. S2. Multiple alignment of nucleotide sequences of the coding region (genomic version) of the pyrG genes of Penicillium chrysogenum (rubens) Wisconsin 54–1255 (Pc_w, Pc13g04420), Penicillium strain PO212 (PO212) and P. oxalicum (Po, PDE_04166). [file MBT2-9-089-s002.tif]

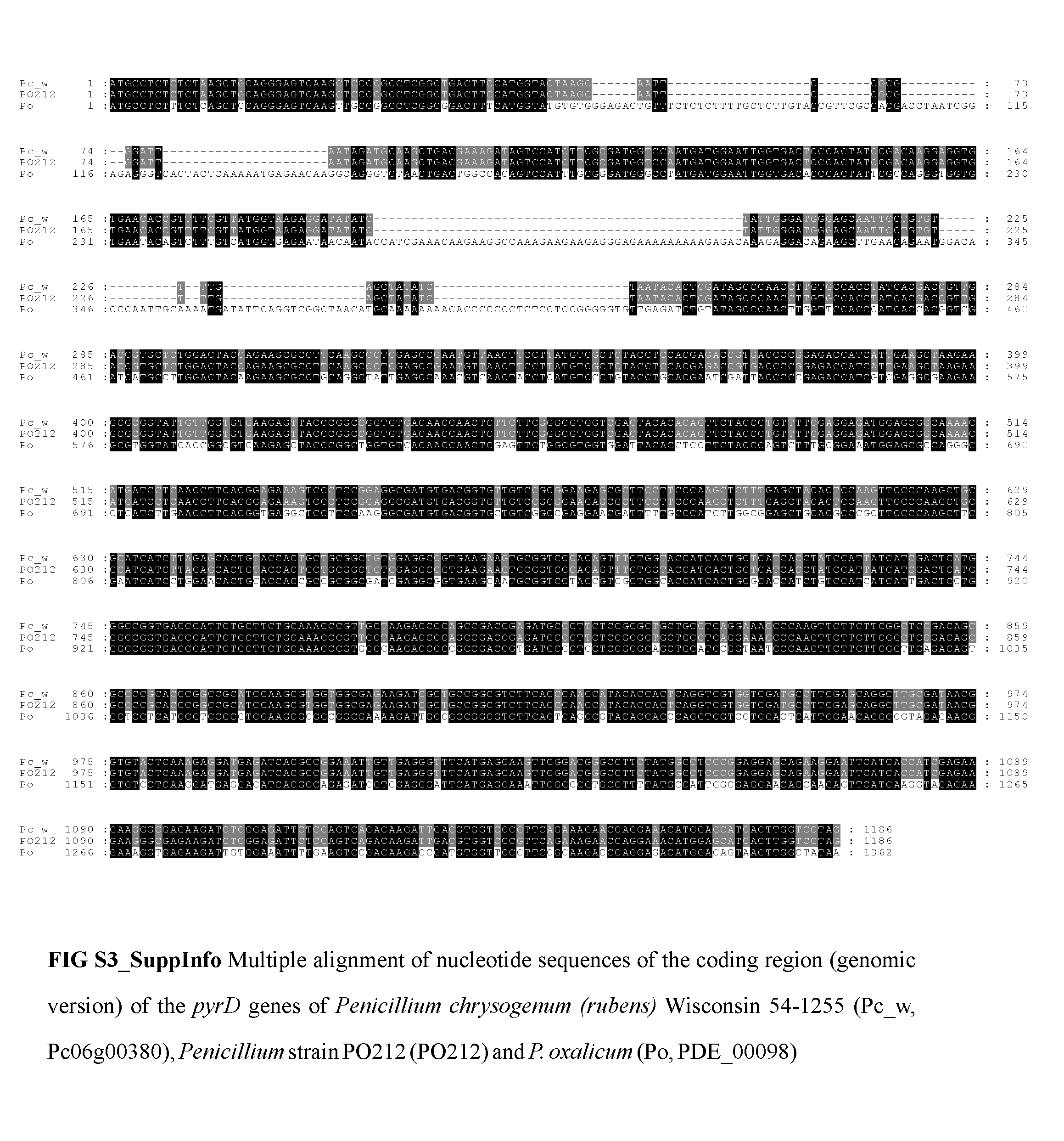

Supplement: Supplementary file 3 — Fig. S3. Multiple alignment of nucleotide sequences of the coding region (genomic version) of the pyrD genes of Penicillium chrysogenum (rubens) Wisconsin 54–1255 (Pc_w, Pc06g00380), Penicillium strain PO212 (PO212) and P. oxalicum (Po, PDE_00098). [file MBT2-9-089-s003.tif]

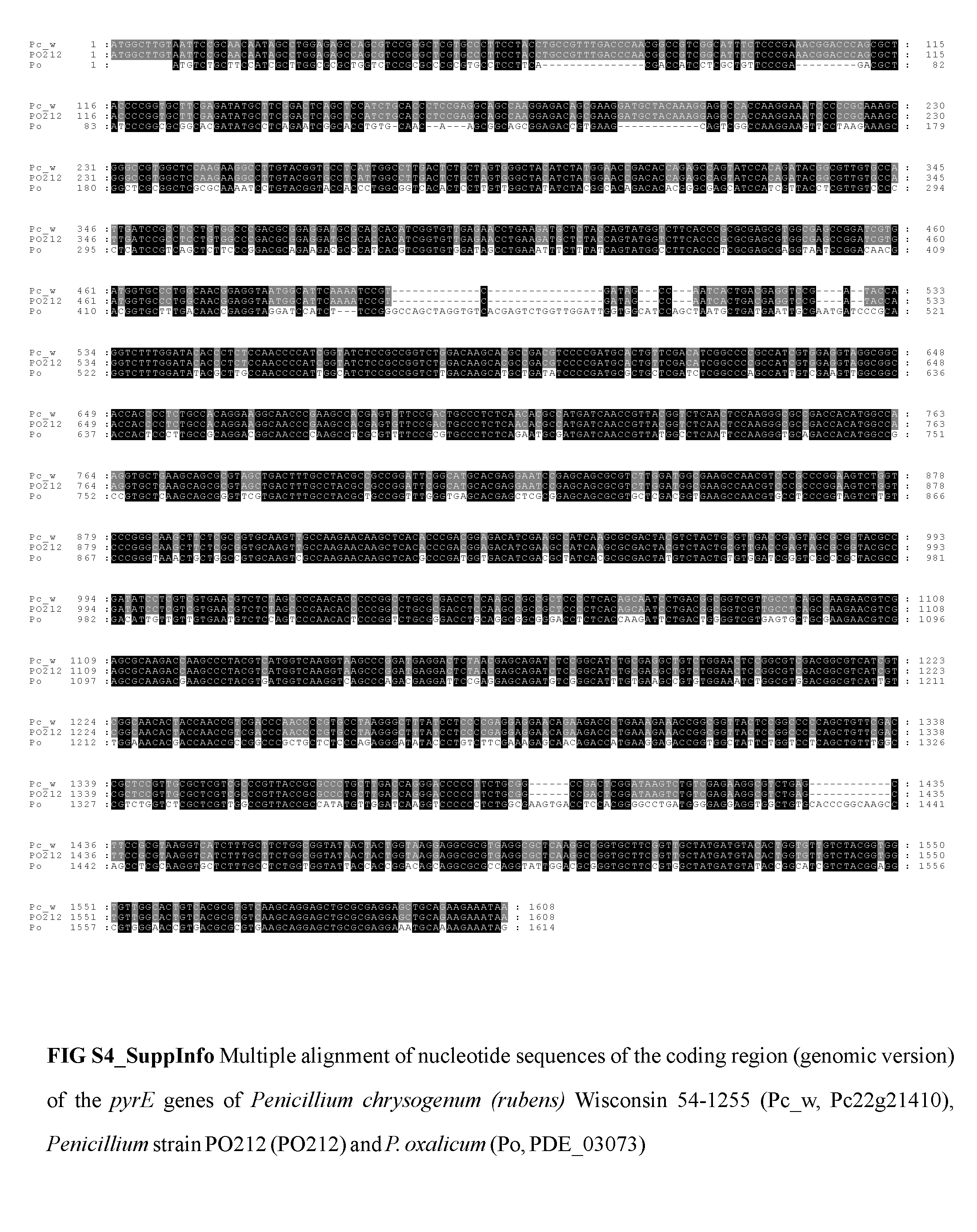

Supplement: Supplementary file 4 — Fig. S4. Multiple alignment of nucleotide sequences of the coding region (genomic version) of the pyrE genes of Penicillium chrysogenum (rubens) Wisconsin 54–1255 (Pc_w, Pc22g21410), Penicillium strain PO212 (PO212) and P. oxalicum (Po, PDE_03073). [file MBT2-9-089-s004.tif]
